# Clinical Evaluation of an Auto-Segmentation Tool for Spine SBRT Treatment

**DOI:** 10.3389/fonc.2022.842579

**Published:** 2022-03-14

**Authors:** Yingxuan Chen, Yevgeniy Vinogradskiy, Yan Yu, Wenyin Shi, Haisong Liu

**Affiliations:** Department of Radiation Oncology, Sidney Kimmel Medical College, Thomas Jefferson University, Philadelphia, PA, United States

**Keywords:** spine SBRT, auto-segmentation, target delineation, clinical target volume (CTV), gross tumor volume (GTV)

## Abstract

**Purpose:**

Spine SBRT target delineation is time-consuming due to the complex bone structure. Recently, Elements SmartBrush Spine (ESS) was developed by Brainlab to automatically generate a clinical target volume (CTV) based on gross tumor volume (GTV). The aim of this project is to evaluate the accuracy and efficiency of ESS auto-segmentation.

**Methods:**

Twenty spine SBRT patients with 21 target sites treated at our institution were used for this retrospective comparison study. Planning CT/MRI images and physician-drawn GTVs were inputs for ESS. ESS can automatically segment the vertebra, split the vertebra into 6 sectors, and generate a CTV based on the GTV location, according to the International Spine Radiosurgery Consortium (ISRC) Consensus guidelines. The auto-segmented CTV can be edited by including/excluding sectors of the vertebra, if necessary. The ESS-generated CTV contour was then compared to the clinically used CTV using qualitative and quantitative methods. The CTV contours were compared using visual assessment by the clinicians, relative volume differences (RVD), distance of center of mass (DCM), and three other common contour similarity measurements such as dice similarity coefficient (DICE), Hausdorff distance (HD), and 95% Hausdorff distance (HD95).

**Results:**

Qualitatively, the study showed that ESS can segment vertebra more accurately and consistently than humans at normal curvature conditions. The accuracy of CTV delineation can be improved significantly if the auto-segmentation is used as the first step. Conversely, ESS may mistakenly split or join different vertebrae when large curvatures in anatomy exist. In this study, human interactions were needed in 7 of 21 cases to generate the final CTVs by including/excluding sectors of the vertebra. In 90% of cases, the RVD were within ±15%. The RVD, DCM, DICE, HD, and HD95 for the 21 cases were 3% ± 12%, 1.9 ± 1.5 mm, 0.86 ± 0.06, 13.34 ± 7.47 mm, and 4.67 ± 2.21 mm, respectively.

**Conclusion:**

ESS can auto-segment a CTV quickly and accurately and has a good agreement with clinically used CTV. Inter-person variation and contouring time can be reduced with ESS. Physician editing is needed for some occasions. Our study supports the idea of using ESS as the first step for spine SBRT target delineation to improve the contouring consistency as well as to reduce the contouring time.

## Introduction

Bone is a frequent site of metastases and causes significant morbidities including severe pain and spinal cord compression (1–3). Stereotactic body radiotherapy (SBRT) has been increasingly used to provide a treatment option in the multidisciplinary management of metastases located within or adjacent (paraspinal) to vertebrae/spinal cord. In SBRT treatments, high dose will be prescribed in typically one to five fractions. Localization accuracy can be managed at millimeter levels with advances in patient immobilization, target visualization, and image-guidance technology (4–7). Target segmentation accuracy becomes critical for spine SBRT due to the requirement of ablative high dose per fraction to the target volume and minimizing the dose to organ at risks, especially the spinal cord. To standardize the target delineation, consensus guidelines for the target volume were published in 2012 for appropriate target volume definition (8). However, manual contouring is time-consuming and has large inter-observer variance. To improve the efficiency and reduce the inter-observer variance, auto-segmentation tools have been developed mainly in three categories: threshold-based methods (9, 10), atlas-based methods (11, 12), and deep learning methods (13, 14). Some methods require human intervention or the manual setting of parameters. Deep learning methods such as supervised learning might be a solution for fully automated spine auto-segmentation, but large training sets are needed. Moreover, based on the International Spine Radiosurgery Consortium (ISRC) Consensus guidelines (8), different anatomical regions (such as vertebral body, pedicles, spinous process, or transverse processes/lamina) will be included in the clinical target volume (CTV) based on location of gross tumor volume (GTV). Most of the above-mentioned published studies are focused on whole spine segmentation, which might not be available to be applied for clinical spine SBRT treatment yet.

Recently, a dedicated software has been developed for spine SBRT treatment (Elements Spine SRS^®^, Brainlab AG, Germany) including auto-segmentation, image fusion, and treatment planning.

Previous evaluation studies have shown the advances of Elements Spine SRS in dosimetry (15–17). Moreover, the auto-segmentation tool Elements SmartBrush Spine (ESS) was developed for fast target delineation, which can potentially improve the efficiency of the clinical workflow. Giaj−Levra et al. have demonstrated that the inter-observer difference can be reduced by using ESS (18) by evaluating the GTV contours. The aim of this study is to evaluate the accuracy of the CTV auto-segmentation based on existing GTV contours using ESS for spine SBRT patients.

To evaluate the performance of the auto-segmentation, analysis metrics were developed to evaluate medical image segmentation (19). Among these, Dice similarity coefficients (DICE) and Hausdorff distance (HD) are common metrics to efficiently evaluate the quality of segmentation. In this study, evaluation metrics including volume differences, distance of center of mass, DICE and HD were selected for the spine segmentation evaluation based on the target contour impact on radiation delivery.

## Materials and Methods

### Patients and Treatments

This study was approved by the Institutional Review Board. Eligible patients required metastasis limited to one vertebral level and without severe compression fracture (loss vertebral height more than 50%). A total of 51 spine SBRT cases treated in our institution from 2018 to 2021 were reviewed. Twenty-one of the 51 cases met this inclusion criterion, and the CTV could be successfully segmented and were evaluated in this retrospective comparison study. Details are shown in **Table 1**. The GTV of 21 targets (12 T spine and 9 L spine) were drawn by physicians based on the MR images and were used as input for the CTV auto-segmentation. The CTV was auto-segmented by ESS on CT scans in two steps. In the first step, the affected vertebra including 6 different sectors were auto-segmented using ESS, which is an atlas-based auto-contouring method. This spine segmentation and labeling of spinal structures in the background enables the automatic CTV calculation. Then, the CTV was generated based on the GTV involvement following the rules from the ISRC guidelines (8). After reviewing the initial target contour, a physician reviewed the auto-segmented CTV and edited the CTV by including/excluding different sectors of the vertebra, if needed, using patient-specific clinical judgment, by simple mouse clicking on each sector. After CTV generation, the planning target volume (PTV) was calculated with uniform expansion with 2 mm margin and modified to avoid potential overlap with the cord. Prescription dose and fraction were determined based on the tumor volume, previous radiation treatment, and surrounding organ-at-risk (OAR) dose tolerance limits.

**Table 1:** Summary of all cases (21 lesions) sorted by clinically used CTV volume.

Case number	Treatment site	CTV volume (cc)	Prescription dose (Gy)	Fractions
**1**	T8	3.95	20	1
**2**	T6	12.3	16	2
**3**	T5	13.7	27	3
**4**	T4	13.8	16	2
**5**	T5	15	18	1
**6**	T11	17.1	16	1
**7**	T9	17.6	18	1
**8**	T9	19.3	24	3
**9**	T5	20.9	24	3
**10**	T7	27	18	1
**11**	T12	28.6	24	3
**12**	L1	39.3	16	1
**13**	L3	45.4	16	1
**14**	L3	45.9	16	1
**15**	L2	46.8	18	1
**16**	T11	48.2	27	3
**17**	L4	49.2	18	1
**18**	T9	50	18	1
**19**	L3	52.4	20	1
**20**	L4	54.4	20	1
**21**	L4	57.4	27	3

### Evaluation Metrics

To evaluate the impact of auto-segmentation for CTV delineation using ESS, the SmartBrush-generated CTVs were compared with the clinically used CTVs using qualitative and quantitative methods. After initial visual assessment and editing by a physician, in-depth quantitative contour comparison metrics were used for comparison, including relative volume difference, distance of center of mass, dice similarity coefficients, structure similarity index measurement, and Hausdorff distance. Both ESS-generated CTVs and clinically used CTVs were exported from Elements as a DICOM file for evaluation. Hausdorff distance was calculated using open source Plastimatch and other evaluation metrics were implemented in MATLAB (The MathWorks, Inc., Natick, MA) using built-in functions. To evaluate the performance statistically, both average and standard deviation (SD) were also calculated for each evaluation metrics.

For the given two different contours A (ESS-generated CTV) and B (clinically used CTV):

Relative volume difference (RVD) is defined as:


$$RVD=\frac{Volume(A)-Volume(B)}{Volume(B)}$$

Distance of center of mass (DCM) is defined as the distance between center of mass of A and B with the unit of mm in this study:


DCM=Dist (Center of A, Center of B)

Dice similarity coefficients (DICE) is defined as:


$$\begin{array}{l} DICE=\frac{2|A\cap B|}{\left|A|+|B\right|} \end{array}$$

DICE is an overlap-based metrics and is widely used for the contour evaluation with a value between 0 to 1. If A and B are exactly the same, then DICE will equal to 1.

Hausdorff distance (HD) is defined as:


HD(A,B)=max{d(A,B),d(B,A)}

Where *d*(*A*, *B*) = *sup*{*d*(*a*, *B*)|*a* ∈ *A*}, *d*(*a*, *B*) = *inf* {*d*(*a*, *b*)|*b* ∈ *B*}, sup represents the supremum, inf represents the infimum. HD is measuring maximum surface distance with the unit of mm in this study. If A and B are exactly the same, then the HD value will equal to 0. As HD is usually sensitive to outliers, 95% Hausdorff distance (HD95) was also calculated. Note that the boundary Hausdorff function in Plastimatch was used to report HD and HD95 in this study.

## Results

**Figure 1:**
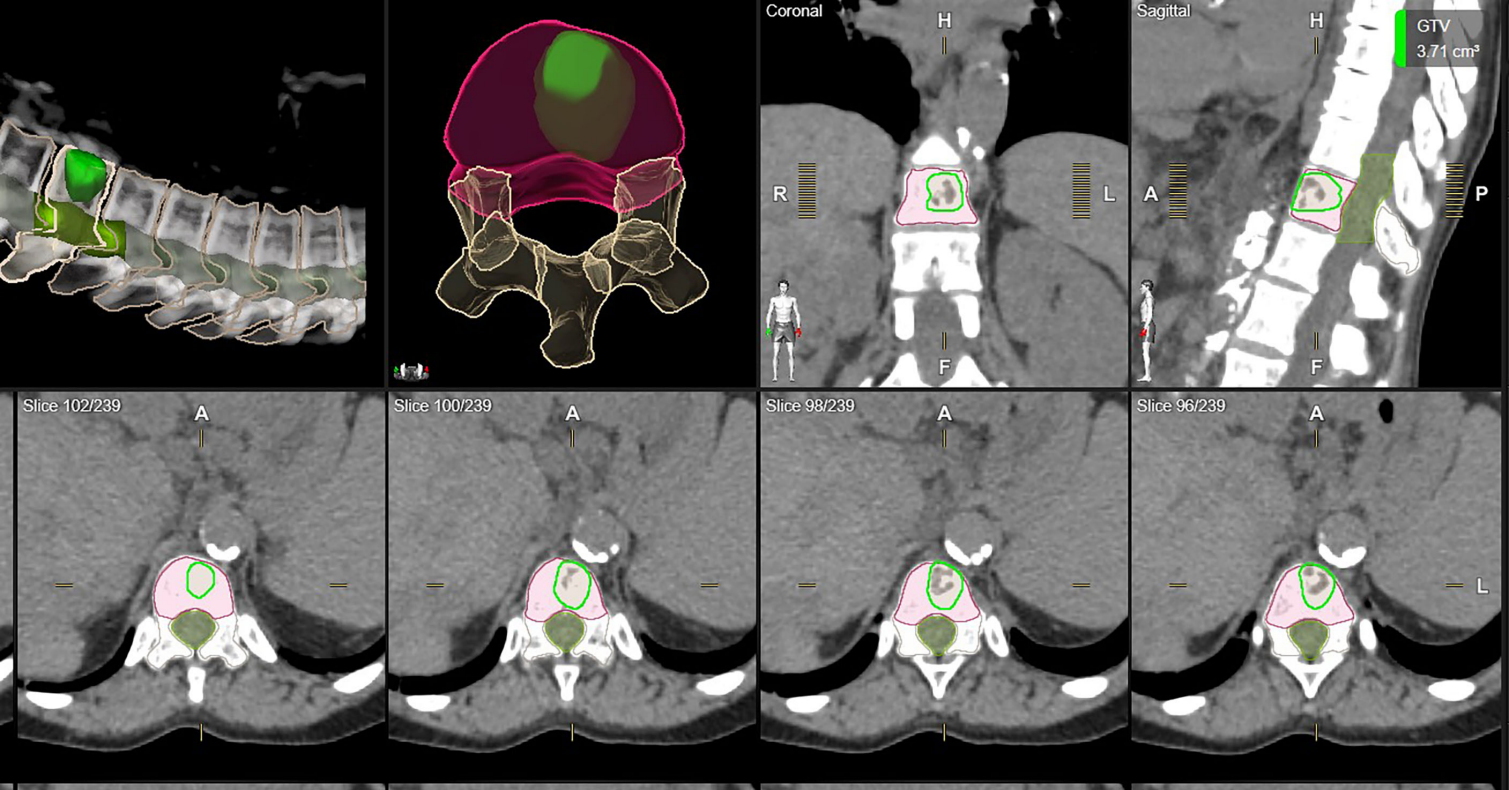
One example of auto-segmented CTV. All vertebrae were segmented and the auto-segmented CTV was automatically generated by ESS following ISRC consensus guidelines. Green: clinically used GTV. Orange: clinically used CTV. Red: ESS-generated CTV, labeled as Clinical Target in Elements.

**Figure 2:**
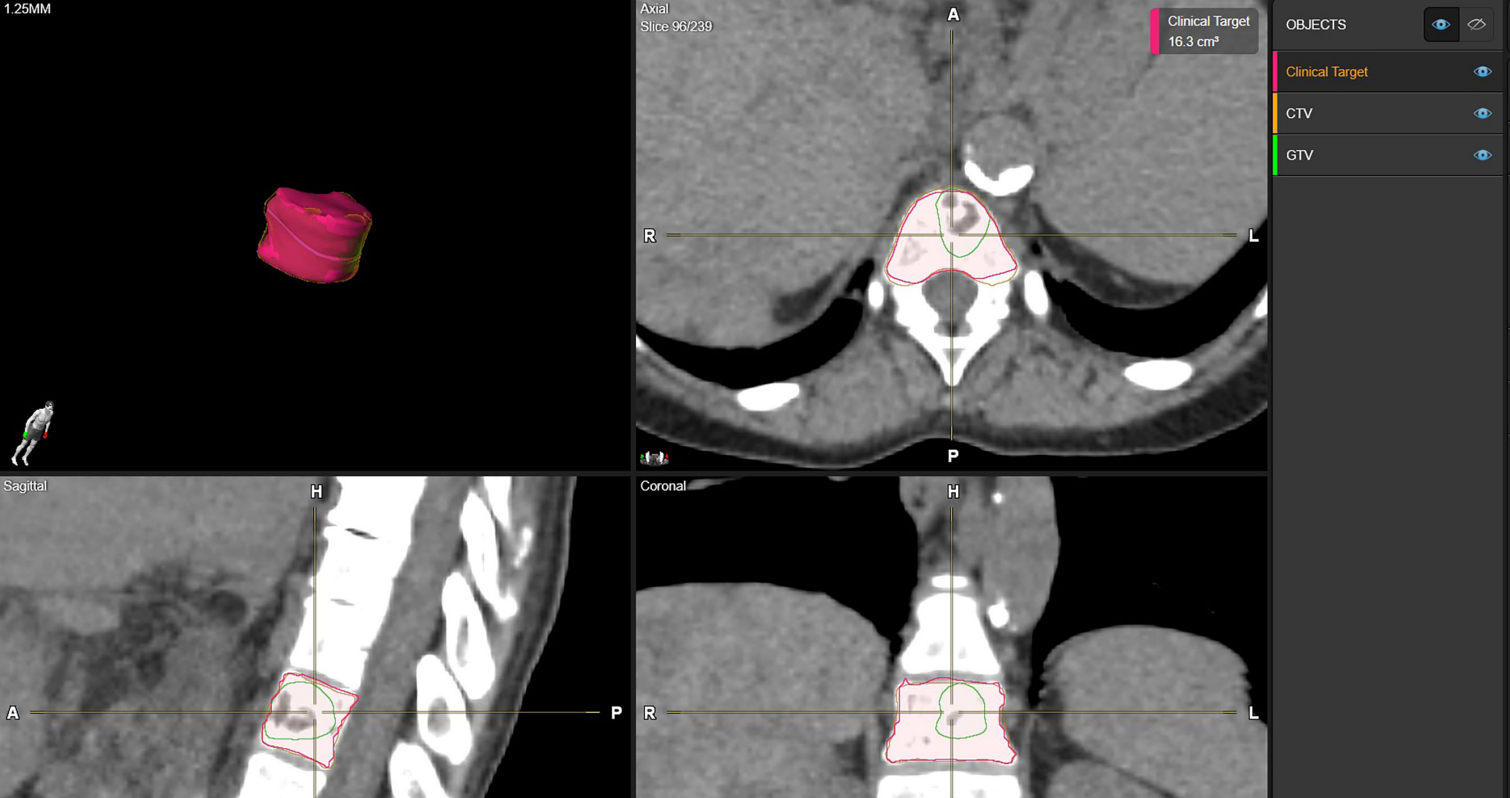
Examples of CTV delineation by manual method and ESS in axial, sagittal, coronal, and 3D views. Green: clinically used GTV. Orange: clinically used CTV. Red: ESS-generated CTV, labeled as Clinical Target in Elements.

**Figure 1** shows examples of vertebral bodies that were auto-segmented by ESS and CTV was generated automatically based on the GTV involvement following ISRC consensus guidelines. A physician reviewed the auto-segmented CTV and edited it by including/excluding different sectors of the vertebra if needed. In this study, 7 of 21 cases need physician’s editing to include/exclude one or more sectors to generate the final CTV. The ESS-generated CTV was labeled as Clinical Target in the software. **Figure 2** shows GTV, ESS-generated CTV, and clinically used CTV of the same patient in 3D and three views (which are axial, sagittal, and coronal). In addition to the 21 cases, there are 30 of 51 reviewed cases that failed the ESS auto-segmentation due to (a) multiple vertebrae (12 cases), (b) paraspinous soft tissue involved in GTV (15 cases), and (c) large curved anatomy like C spine at neck region or L spine and sacrum junction (3 cases). Both a and b situations are not implemented in the current version of ESS auto-segmentation.

Analysis results are summarized in **Table 2**. Average volume of clinically used CTV was 32.3 cc (range from 3.95 cc to 57.4 cc) while ESS-generated CTV ranged from 5.52 cc to 60.9 cc, with an average volume of 33.17 cc. The average of relative volume difference (RVD), distance of center of mass (DCM), and dice similarity coefficient (DICE), Hausdorff distance (HD), and 95% percentile Hausdorff distance (HD95) for the 21 cases were 3% ± 12%, 1.9 ± 1.5 mm, 0.86 ± 0.06, 13.34 ± 7.47 mm, and 4.67 ± 2.21 mm, respectively.

**Table 2:** Summary of comparison between clinically used CTV and auto-segmented CTV regarding volume, DCM, DICE, SSIM, HD, and HD95.

Case number	Clinically used CTV Volume (cc)	SmartBrush-generated CTV Volume (cc)	Absolute Vol Diff (cc)	RVD (%)	DCM (mm)	DICE	HD (mm)	HD95 (mm)
1	3.95	5.52	1.57	42.0%	1.59	0.69	13.55	6.40
2	12.30	14.10	1.80	15.2%	0.73	0.88	6.43	2.77
3	13.70	13.70	0.00	−0.1%	0.66	0.82	9.17	4.18
4	13.80	12.80	−1.00	−7.6%	0.39	0.88	6.25	2.50
5	15.00	14.00	−1.00	−6.7%	1.78	0.87	5.75	2.52
6	17.10	16.30	−0.80	−3.0%	1.06	0.94	2.83	1.27
7	17.60	17.00	−0.60	−1.4%	1.54	0.70	20.80	8.78
8	19.30	17.60	−1.70	−7.8%	2.90	0.89	7.43	2.54
9	20.90	18.80	−2.10	−9.8%	4.17	0.86	7.71	3.75
10	27.00	27.70	0.70	1.9%	1.14	0.84	17.31	4.79
11	28.60	27.00	−1.60	−5.7%	1.59	0.83	21.50	6.94
12	39.30	39.40	0.10	0.9%	0.60	0.94	4.38	2.50
13	45.40	51.70	6.30	13.5%	4.21	0.86	19.52	4.51
14	45.90	47.10	1.20	2.9%	1.63	0.88	23.10	5.60
15	46.80	55.90	9.10	20.4%	0.72	0.86	26.84	9.04
16	48.20	49.07	0.87	1.8%	6.04	0.87	15.84	4.48
17	49.20	47.30	−1.90	−4.1%	0.61	0.87	21.47	6.67
18	50.00	50.50	0.50	1.0%	1.80	0.89	9.81	4.70
19	52.40	56.40	4.00	7.5%	1.50	0.91	6.41	2.65
20	54.40	53.70	−0.70	−1.5%	1.21	0.89	9.02	3.19
21	57.40	60.90	3.50	7.0%	3.65	0.83	24.97	8.24
**Average**	**32.30**	**33.17**	**0.87**	**3.0%**	**1.90**	**0.86**	**13.34**	**4.67**
**SD**	**17.28**	**18.61**	**2.83**	**12.0%**	**1.50**	**0.06**	**7.47**	**2.21**

The bold values are Average and SD to distinguish from values of each case.

**Figure 3:**
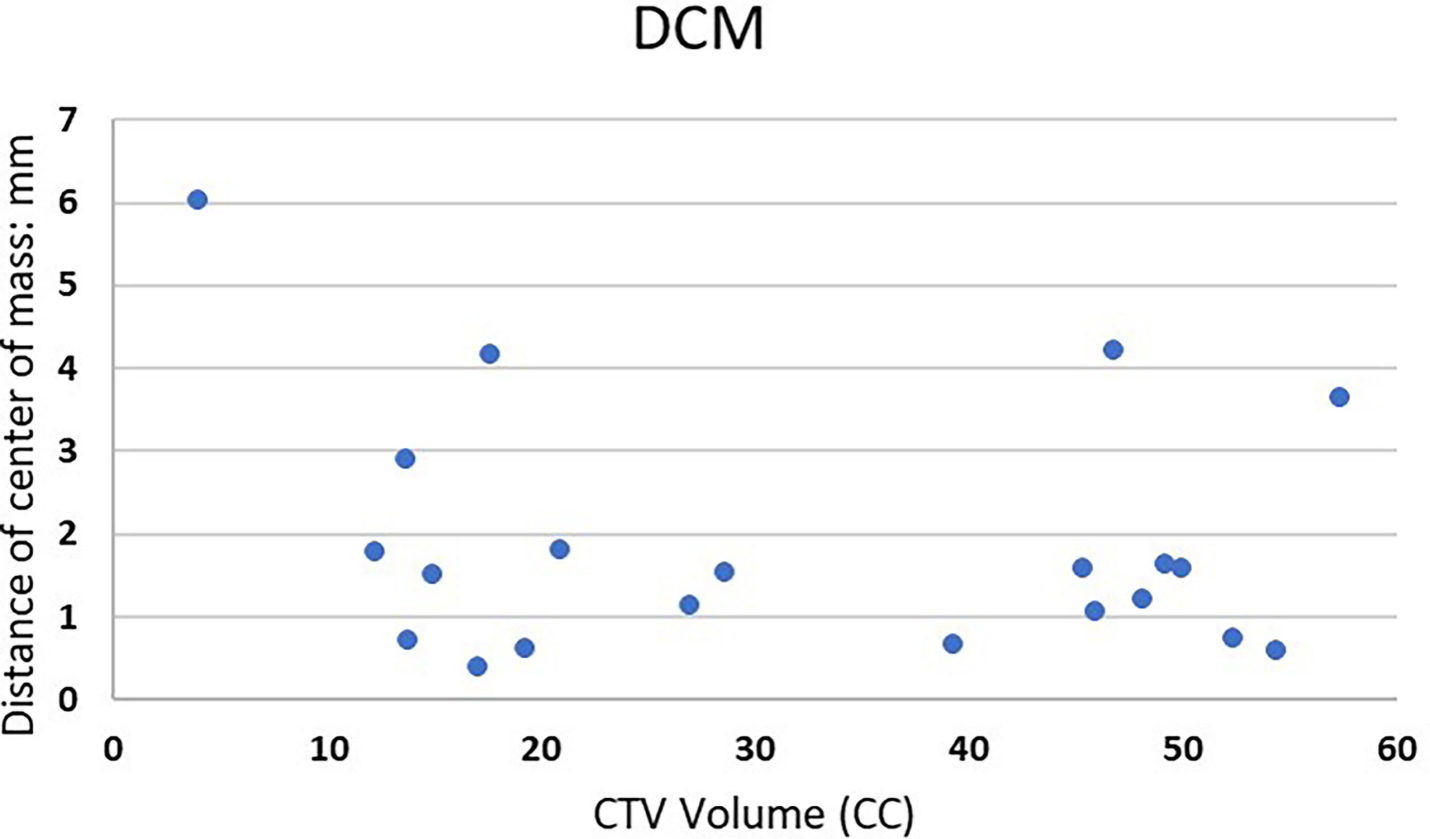
Distance of center of mass of 21 lesions vs. clinically used CTV volume.

**Figure 3** shows DCM for the 21 lesions as a function of clinically used CTV volume. For the treatment planning, the center of the target was usually selected as the treatment isocenter. The DCM was below 2 mm for 16 out of the 21 cases.

**Figure 4:**
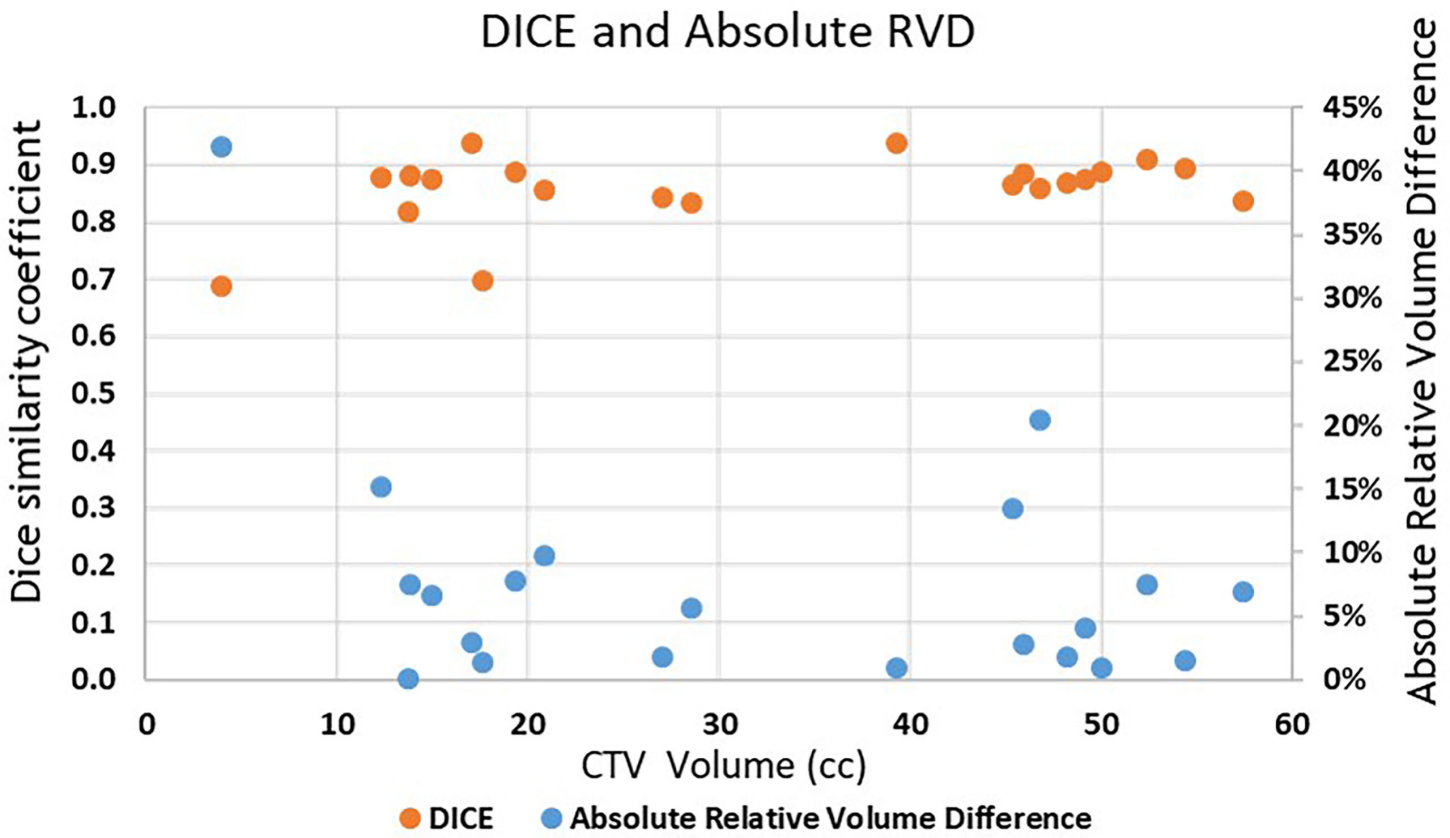
Absolute RVD and DICE of the 21 lesions vs. clinically used CTV volume. Blue dots are Absolute RVD and orange dots are DICE.

Absolute RVD and DICE are shown in **Figure 4**. Both absolute RVD and DICE are volume-based evaluation metrics. Low relative volume is associated with high DICE, which indicate good agreement between ESS-generated CTV and clinically used CTV.

## Discussion

**Figure 5:**
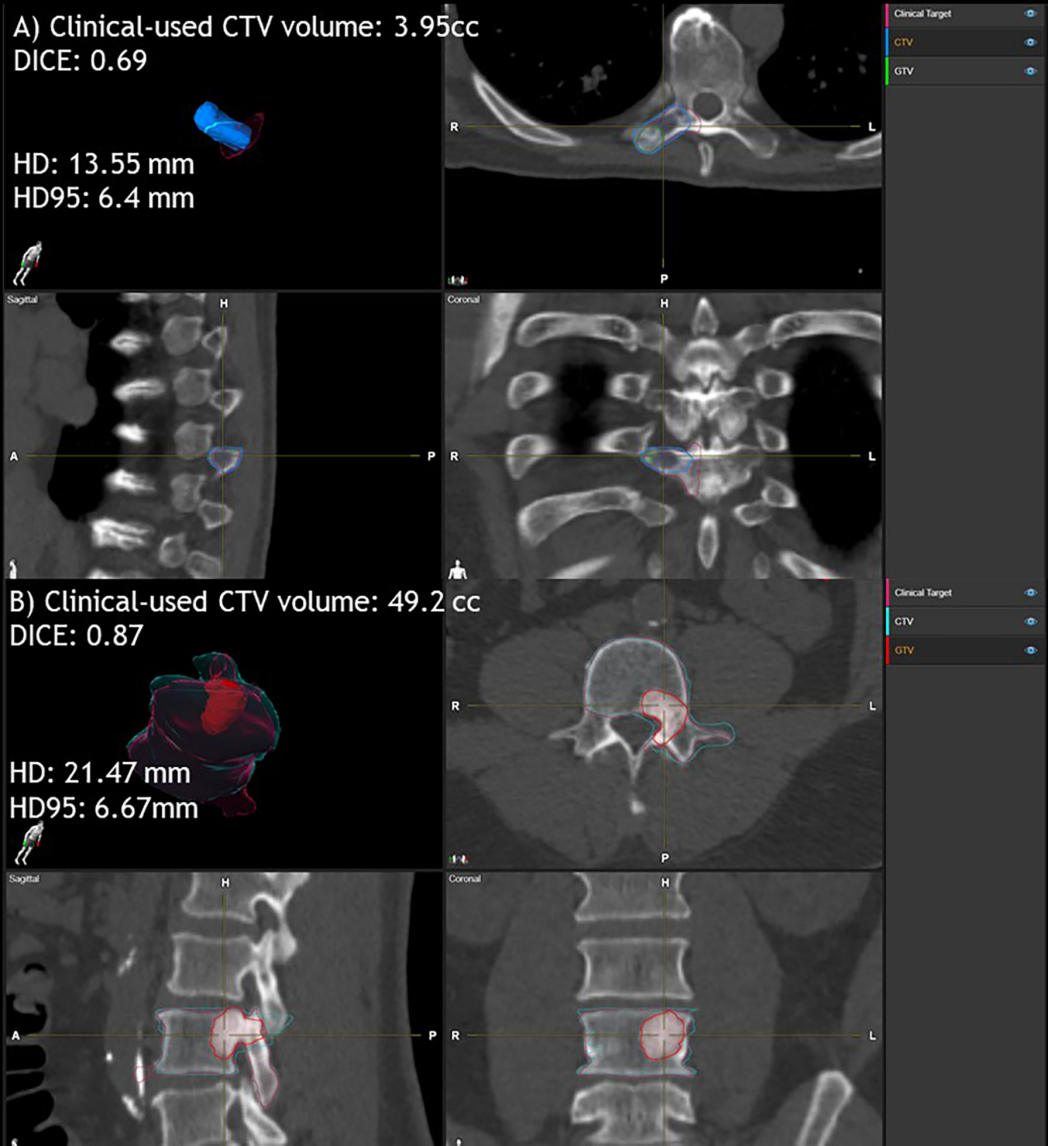
Examples of clinically used CTV and ESS-generated CTV comparison for small and large volumes. Case **(A)** clinically used CTV volume is 3.95 cc. DICE is 0.69, HD is 13.55 mm and HD95 is 6.4 mm. Case **(B)** clinically used CTV volume is 49.2 cc. Dice is 0.87, HD is 21.47 mm and HD95 is 6.67 mm. Here, ESS-generated CTV is labeled as Clinical Target.

Accurate target delineation has significant impact on the quality of the radiation treatment plan. For a spine SBRT approach, correct definition of the treatment volume becomes even more important due to the nature of this treatment with high dose delivery per fraction and the proximity of critical OARs such as the spinal cord. Many studies have already demonstrated that inter-observer variability can be reduced by using the auto-segmentation tool (18). Our study also supports the findings. Overall, the ESS-generated CTVs have a good agreement with the clinically used CTVs. Different evaluation metrics can display the similarity in different aspects. It is highly recommended to use multiple metrics to evaluate contours in different aspects. For example, DICE is a volume-based evaluation metric that might be less sensitive to evaluate large volume contours. Otherwise, Hausdorff distance measures the surface distance between two contours, which can be used in the contour shape evaluation. For the contour with a small volume, even they have large relative volume differences, and HD and HD95 might not be large as shown in **Figure 5A**. In contrast, as shown in **Figure 5B**, high DICE cases might also have high HD and HD95 depending on the shape of the contours with outliers of large surface distances.

After reviewing each case, clinically used CTV is more likely to include inter-space between vertebrae more generously if the spine is not parallel to the axial CT slices. ESS splits the vertebra into 6 sectors according to the ISRC consortium guidelines. The accuracy of identifying the vertebra as well as sectors of vertebral body can be improved significantly if the auto-segmentation is used as the first step. Moreover, the users have the flexibility of clicking and selecting to include or exclude different sectors, after reviewing the auto-segmented CTV, which will potentially improve the efficiency of the clinical workflow.

For most T or L spine cases, ESS can be an efficient tool to automatically generate CTVs on CT images based on the GTV locations. As discussed in the method session, 21 of the 51 cases met the inclusion criteria and the CTV could be successfully segmented and were evaluated in this study. For the other cases, failed auto-segmentation was due to some limitations for the current version. During the evaluation, we observed that ESS failed to segment a CTV if (a) the GTV involves multiple vertebrae (12 cases) or (b) paraspinous soft tissue was involved in the treatment target (15 cases). In addition, it might be challenging to segment CTV for C spine (1 case) at the neck region, or the L spine and sacrum junction (2 cases) and spine might be split mistakenly using ESS when large curved anatomy relative to the CT slices exists. Therefore, careful physician review and confirmation is needed.

There are some limitations of this study. Firstly, only 21 cases were in-depth evaluated and the small sample size may introduce some statistical bias. Only CTVs were evaluated in this study. Surrounding OARs contouring accuracy is also important for plan optimization and evaluation. In the future, both CTV and OAR analysis for larger samples or cross-institutions could be potentially carried out.

## Conclusion

Elements SmartBrush Spine can auto-segment a CTV quickly and accurately and has good agreement with the clinically used CTV. Inter-person variation can be reduced with ESS. Physician editing is needed for some occasions. Our study supports the idea of using ESS as the first step for spine SBRT target delineation to improve the contouring consistency as well as to reduce contouring time, which might potentially improve the efficiency and precision of the spine SBRT treatment.

## Author’s Note

Part of the study will be presented at the Radiosurgery Society (RSS) Scientific Meeting in 2022.

## Data Availability Statement

The raw data supporting the conclusions of this article will be made available by the authors, without undue reservation.

## Ethics Statement

The studies involving human participants were reviewed and approved by Office of Human Research, Institutional Review Board (IRB) of Thomas Jefferson University. Written informed consent for participation was not required for this study in accordance with the national legislation and the institutional requirements.

## Author Contributions

YC, HL, and WS contributed to conception and design of the study. HL performed the data collection. YC developed evaluation tools and performed the analysis. YC wrote the first draft of the manuscript. All authors contributed to manuscript revision, read, and approved the submitted version.

## Conflict of Interest

Thomas Jefferson University has a research agreement with Brainlab AG for evaluation of Elements SmartBrush Spine technology, for which HL and WS are the principal investigators. The funder was not involved in the study design, data collection, analysis, and interpretation, the writing of this article, or the decision to submit it for publication. In addition, WS received consulting fee from Varian, Brainlab, Novocure, and Zai lab, and research funding from Brainlab, Novocure, and regeneron. YV received 2 NCI grants (UG3CA247605 and R01CA236857) and a grant from MIM Software. None of these are related to this research work.

The remaining authors declare that the research was conducted in the absence of any commercial or financial relationships that could be construed as a potential conflict of interest.

## Publisher’s Note

All claims expressed in this article are solely those of the authors and do not necessarily represent those of their affiliated organizations, or those of the publisher, the editors and the reviewers. Any product that may be evaluated in this article, or claim that may be made by its manufacturer, is not guaranteed or endorsed by the publisher.
